# Supplementary material for: Terrorist attacks sharpen the binary perception of “Us” vs. “Them”
Source: Sci Rep. 2023 Aug 1;13:12451. doi: 10.1038/s41598-023-39035-3 (PMC10394060; doi:10.1038/s41598-023-39035-3)
Supplement: Supplementary file 1 — Supplementary Information. [file 41598_2023_39035_MOESM1_ESM.pdf]

Appendix A Details of our sample of terrorist attacks

Date	Wiki article (abbreviation)	Details	Deaths	Injuries
13-Nov-15	November 2015 Paris attacks[1] (Paris 2015)	A series of co-ordinated attacks. The first shooting attack occurred in a restaurant and a bar in the 10th arrondissement of Paris. Other bombings took place outside the Stade de France stadium in the suburb of Saint-Denis.	130 (+7 attackers)	413
22-Mar-16	2016 Brussels bombings[2] (Brussels 2016)	There were three coordinated suicide bombings in Brussels: two at Brussels Airport in Zaventem, and one at Maalbeek metro station.	32 (+3 attackers)	340
13-Jun-16	2016 Magnanville stabbing[3] (Magnanville 2016)	A man stabbed and killed a police officer in his home, before taking the officer's wife and son hostage. ISIL claimed responsibility.	2 (+1 attacker)	0
14-Jul-16	2016 Nice attack[4] (Nice 2016)	A cargo truck was deliberately driven into crowds celebrating Bastille Day on the Promenade des Anglais in Nice. ISIL claimed the responsibility.	86 (+1 attacker)	458
19-Dec-16	2016 Berlin attack[5] (Berlin 2016)	A truck was driven into a Christmas market in Berlin. ISIL claimed responsibility.	12	56
22-Mar-17	2017 Westminster attack[6] (Westminster 2017)	A Muslim convert drove a car into pedestrians on Westminster Bridge, He then crashed his car into the fence of the Palace of Westminster and fatally stabbed an unarmed policeman.	5 (+1 attacker)	50
03-Apr-17	2017 Saint Petersburg Metro bombing[7] (St Petersburg 2017)	A suicide bomber blew himself up on the St Petersburg Metro, Imam Shamil Battalion, an Al-Qaeda affiliate, claimed responsibility, but according to the FSB, attacker acted on the orders of a field commander from ISIL.	5 (+1 attacker)	64
07-Apr-17	2017 Stockholm attack[8] (Stockholm 2017)	A hijacked truck was driven into pedestrians along a shopping street before crashing into a department store. The attacker had shown sympathies for extremist organizations including ISIL.	5	14
22-May-17	Manchester Arena bombing[9] (Manchester 2017)	A suicide bombing was carried out at Manchester Arena after a concert by American singer Ariana Grande.	22 (+1 attacker)	512
03-Jun-17	2017 London Bridge attack[10] (London Bridge 2017)	A van ran into pedestrians on London Bridge and then drove to Borough Market.	8 (+3 attackers)	48
16-Aug-17	2017 Barcelona attacks[11] (Barcelona 2017)	Two suspects were killed in an initial accidental explosion during the preparation of explosives. Latter on, a van was driven into pedestrians in Las Ramblas, Barcelona. The following day a related attack occurred in Cambrils. ISIL claimed responsibility.	16 (+8 attackers)	152
18-Aug-17	2017 Turku stabbing[12] (Turku 2017)	An ISIL inspired attacker said a motive for his attack was airstrikes by the Western Coalition during the 2017 Battle of Raqqa in Syria.	2	8 (+1 attacker)
01-Oct-17	Marseille stabbing[13] (Marseille 2017)	Two women were stabbed by a migrant from Tunisia. ISIL claimed responsibility.	2 (+1 attacker)	0
23-Mar-18	Carcassonne and Trèbes attack[14] (Carcassonne 2018)	An attacker stole a car, killing a passenger in Carcassonne. Then, he attacked a supermarket.	4 (+1 attacker)	15
29-May-18	2018 Liège attack[15] (Liege 2018)	A man killed two police officers and a civilian. He is also believed to have killed a man the day before the attack.	4 (+1 attacker)	4

Overview and descriptions of 15 terrorist attacks comprising our sample and the abbreviations used in the main text. Dates are as established for our analysis, although some attacks spanned several days.

Appendix B Arrangement of surviving 69 articles into Specific and Broad categories

Broad category	Specific category	Article from English Wikipedia
ENEMY	Terrorism in general	Massacre
		Mass shooting
		Lone wolf (terrorism)
	Islamic terrorism	2002 Los Angeles International Airport shooting
		2004 SuperFerry 14 bombing
		2007 Glasgow Airport attack
		2010 Moscow Metro bombings
		Abduction of Russian diplomats in Iraq
		List of terrorist incidents linked to ISIL
		Muslim Brotherhood
		Rick Leventhal
		Kimberly Dozier
	Non-Islamic terrorism	Basque conflict
		Basque National Liberation Movement
		Timeline of Real Irish Republican Army actions
	Terrorism in Western countries	List of terrorist incidents in Australia
		List of terrorist incidents in Great Britain
		List of terrorist incidents in London
		Terrorism in Europe
		Terrorism in Greece
		Terrorism in Sweden
		Terrorism in the Philippines
	Conspiracies	False flag
SECURITY	National security	Conscription in the United States
		Crisis actor
		List of police firearms in the United Kingdom
		Police use of firearms in the United Kingdom
		Ring of steel (London)
		Spanish Armed Forces
		UK Threat Levels
		Universal background check
		Devin Nunes
	Weapons	Comparison of the AK-47 and M16
		Gun culture in the United States
		Gun politics in the United States
		Jews for the Preservation of Firearms Ownership
		Assault weapon
		USS Cheyenne (SSN-773)
		United States v. Miller
	Transport options	Big Sky Airlines
		Boston-Maine Airways
		Corporate Air
		Dakota, Minnesota and Eastern Railroad
		Eclipse Aviation
	Insurance options	Thornburg Mortgage
		TD Banknorth
	Equipment for survival	Orvis
OTHER PERCEPTION	Islam in general	Moinuddin Chishti
	Islam in Western countries	Islam in Poland
		List of mosques in the United Kingdom
		Ed Husain
		Bhagwan Shree Rajneesh
	Islam in non-Western countries	Islam in Qatar
	About Middle East	Susa
SELF PERCEPTION	National identity	Juan Carlos I
	Religious identity	Church of the Holy Sepulchre
	Activism in general	Kyle Kulinski
		Andrew O'Hagan
	Anti-Islam activism	James Wickstrom
		Quilliam (think tank)
		Tommy Robinson (activist)
		Sebastian Gorka
		Paul Weston (politician)
	Violent history of Western countries	Grdelica train bombing
		Greek War of Independence
		Legitimacy of the NATO bombing of Yugoslavia
		Charles B. McVay III
		The Man with the Iron Heart
		List of events named massacres

Organization of the 69 articles that survived all filters (right column, same as Table 1) into Specific categories (middle column) and broad categories (left column).

Appendix C Dynamics of total visits to English Wikipedia in relation to the attacks

Do terrorist attacks divert attention from other simultaneously ongoing concerns? Here, we examine the time series of daily viewcounts to all articles cumulatively, excluding only stubs and redirects (see section 4.5, immediately after the exclusion Step 1). The obtained time series is shown in Fig. 1 (top panel), in which we marked our sample of attacks. For better clarity, in the bottom panel of Fig. 1 we zoom to a 3-month period in 2017 during which five attacks occurred.

The total views vary from 200 to 300 million per day. There are pronounced and stable weekly oscillations, with more activity during weekdays and less during the weekend. There are less pronounced seasonal oscillations, with slightly more activity during the early months of the year (winter in the northern hemisphere). As the Internet becomes ever more accessible, we expected to find more viewing activity in 2018 compared to 2015. Surprisingly, we found none.

None of the considered terrorist events seems to affect the overall viewing dynamics, as clear from the bottom panel of Fig. 1. Weekly oscillations remain statistically stable immediately after any of the attacks. In the eyes of the global English-speaking public, terrorism is only one of very many concerns ongoing in parallel. Attacks might have diverted some attention from routine concerns, but the intensity of this diversion is too small to be recognized against the background of normal fluctuations.

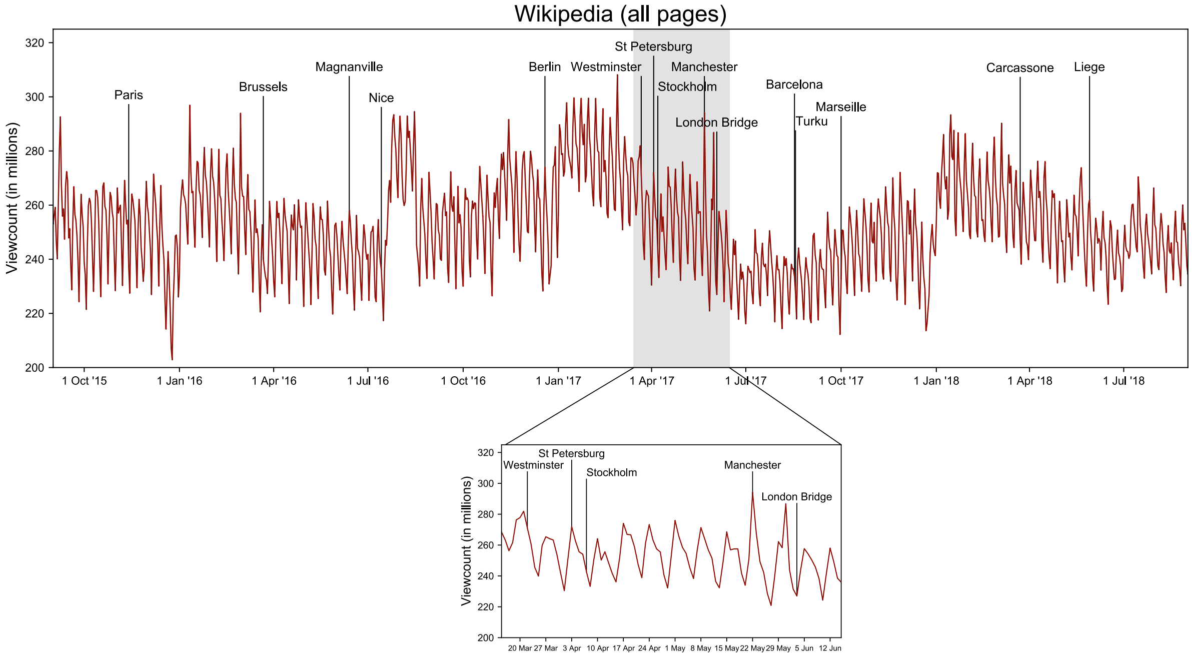

Figure 1: The time series of daily viewcounts to all articles in English Wikipedia excluding only stubs and redirects. Top panel: the time series with dates of the attacks shown. Bottom panel: the same time series for 3 months in 2017 during which five attacks occurred (dates shown on x-axis are all Mondays).

Appendix D Bench-marking against reactions to other events

What else, besides terrorist attacks, diverts public attention to these issues? We investigate this by systematically computing Excess Attention Z for all articles from Table 1, starting with the Paris 2015 event. For each article, we isolate the reactions with $Z > 3$ that happened at least two weeks away from any of the attacks. This led to a list of non-terrorism-related reactions for each article.

We looked for patterns in this list but found none to be very clear. For a typical article, we found about 10–30 reactions to events other than terrorism. This number seems relatively constant across all broad categories. Overall, articles with more general content (e.g. *Terrorism in Europe*) had more, and articles with more focused contents (e.g. *Terrorism in Greece*) had less non-terrorism-related reactions. While broad categories react (in specific ways) to acts of terrorism, they also react to other events, albeit in a less consistent manner. This comes across as natural, since terrorism is not the only public concern. For instance, we found the viewcount of several articles from Table 1 to spike on 25 January 2017. Yet many events could have been responsible: the inauguration of US President Donald Trump, withdrawal of the US from the Trans-Pacific Partnership, Oscar nominations, Syrian civil war, etc.

Along the same lines, we checked what else draws attention to the 15 articles devoted to the attacks themselves. To this end, we looked for large Z away from all attacks. We found a few non-terrorism-related reactions, but much less than for articles from Table 1. It appears that the best 'markers' of terrorist attacks are articles devoted to the attacks themselves. At any rate, the message of the above analysis is that too many events occur in the world simultaneously for any clear-cut result on non-terrorism-related reactions.

Appendix E Robustness checks

Critical for reaching our results was the choice of exclusion criteria. Indeed, many of our choices may appear arbitrary, leading one to think that different exclusion choices could have led to different results. For this reason, we checked whenever possible the robustness of the results against variations of the exclusion criteria. Upon very close examination we found no significant impact of such variations on our results, i.e., the overall picture (such as the structure of broad categories) still holds even with non-trivial modifications of our exclusion choices.

In the same context, recall that in defining Excess Attention, we implicitly assumed that daily viewcounts (in fact, their weekly averages) are normally distributed around the mean, which may not always be the case. But in practice, as clear from Fig. 9, $Z > 3$ is a sufficient guarantee for a redirection of attention, even if viewcounts are not (perfectly) normally distributed. Upon excluding articles with $Z < 3$, the only false positives we found (and examined manually, see 4.5) were the issues that accidentally coincided with the attacks. This confirms the validity of our $Z > 3$ criterion regardless of viewcount distribution.

On the other hand, our results could benefit from an independent verification via sources other than Wikipedia. These would rely primarily on social media, which nowadays are a standard channel of communication. This is an important direction for future work since it can elucidate the public reaction from a wider perspective. Still, each tweet is just one particular message (usually) designed by one person. Even if this tweet is very popular, its popularity might be due to a lack of a systematic répertoire of tweets from which the audience could choose the best ones. Alternatively, one could verify our results via surveys and interviews: Ask people directly what interests them after a terrorist attack. However, there is a clear difference between what people do and what they report. Surveys typically measure the reported behavior only. In contrast, our insights are in the actual views of Wikipedia, hence revealing the *real behavior*.

Appendix References

[1] Wikipedia. *November 2015 Paris Attack*. URL: https://en.wikipedia.org/wiki/November_2015_Paris_attacks (visited on 08/08/2019).

[2] Wikipedia. *2016 Brussels bombing*. URL: https://en.wikipedia.org/wiki/2016_Brussels_bombings (visited on 08/08/2019).

[3] Wikipedia. *2016 Magnanville stabbing*. URL: https://en.wikipedia.org/wiki/2016_Magnanville_stabbing (visited on 08/08/2019).

[4] Wikipedia. *2016 Nice truck attack*. URL: https://en.wikipedia.org/wiki/2016_Nice_truck_attack (visited on 08/08/2019).

[5] Wikipedia. *2016 Berlin truck attack*. URL: https://en.wikipedia.org/wiki/2016_Berlin_truck_attack (visited on 08/08/2019).

[6] Wikipedia. *2017 Westminster attack*. URL: https://en.wikipedia.org/wiki/2017_Westminster_attack (visited on 08/08/2019).

[7] Wikipedia. *2017 Saint Petersburg Metro bombing*. URL: https://en.wikipedia.org/wiki/2017_Saint_Petersburg_Metro_bombing (visited on 08/08/2019).

[8] Wikipedia. *2017 Stockholm truck attack*. URL: https://en.wikipedia.org/wiki/2017_Stockholm_truck_attack (visited on 08/08/2019).

[9] Wikipedia. *Manchester Arena bombing*. URL: https://en.wikipedia.org/wiki/Manchester_Arena_bombing (visited on 08/08/2019).

[10] Wikipedia. *2017 London Bridge attack*. URL: https://en.wikipedia.org/wiki/2017_London_Bridge_attack (visited on 08/08/2019).

[11] Wikipedia. *2017 Barcelona attacks*. URL: https://en.wikipedia.org/wiki/2017_Barcelona_attacks (visited on 08/08/2019).

[12] Wikipedia. *2017 Turku attack*. URL: https://en.wikipedia.org/wiki/2017_Turku_attack (visited on 08/08/2019).

[13] Wikipedia. *2017 Marseille stabbing*. URL: https://en.wikipedia.org/wiki/2017_Marseille_stabbing (visited on 08/08/2019).

[14] Wikipedia. *Carcassonne and Trèbes attack*. URL: https://en.wikipedia.org/wiki/Carcassonne_and_Tr%5C%C3%5C%A8bes_attack (visited on 08/08/2019).

[15] Wikipedia. *2018 Liège attack*. URL: https://en.wikipedia.org/wiki/2018_Li%5C%C3%5C%A8ge_attack (visited on 08/08/2019).
